# Whole-body kinematic and dynamic modeling for quadruped robot under different gaits and mechanism topologies

**DOI:** 10.7717/peerj-cs.821

**Published:** 2021-12-16

**Authors:** Wei Yan, Yang Pan, Junjie Che, Jiexian Yu, Zhuchen Han

**Affiliations:** 1 Shenzhen Key Laboratory of Biomimetic Robotics and Intelligent Systems, Department of Mechanical and Energy Engineering, Southern University of Science and Technology, Shenzhen, China; 2 Guangdong Provincial Key Laboratory of Human-Augmentation and Rehabilitation Robotics in Universities, Southern University of Science and Technology, Shenzhen, China

**Keywords:** Whole body dynamic, Screw theory, Quadruped robot, Mechanism topologies

## Abstract

Dynamic locomotion plays a crucial role for legged robots to fulfill tasks in unstructured environments. This paper proposes whole-body kinematic and dynamic modeling method s based on screw theory for a quadruped robot using different gaits and mechanism topologies. Unlike simplified models such as centroid or inverse pendulum models, the methods proposed here can handle 10-dimensional mass and inertia for each part. The only simplification is that foot contact models are treated as spherical joints. Models of three different mechanism topologies are formulated: (1) Standing phase: a system consisting of one end-effector, the body, and four limbs, the legs; (2) Walking phase: a system consisting of one or two lifting legs (depending on the chosen gait), two or three supporting legs; (3) Floating phase: a system in which all legs detach from the ground. Control strategies based on our models are also introduced, which includes walk and trot gait plans. In our control system, two additional types of information are provided: (1) contacting forces are given by force sensors installed under feet; (2) body poses are determined by an inertial measurement unit (IMU). Combined with the sensor data and calibrated mass, inertia, and friction, the joint torque can be estimated accurately in simulation and experiment. Our prototype, the “XiLing” robot, is built to verify the methods proposed in this paper, and the results show that the models can be solved quickly and leads to steady locomotions.

## Introduction

Compared with wheeled and tracked mobile machines, legged robots have apparent advantages when working in unstructured environments. In the past few decades, remarkable improvements have been witnessed in the agility and mobility of legged robots. For instance, the biped Atlas (Kuindersma et al., 2016) and quadruped BigDog (Raibert et al., 2008), both actuated by hydraulic systems, and the quadruped spot mini driven by electric motors have demonstrated their capabilities in highly complex motions. In addition, the quadruped HyQ designed by Semini et al. (2011), which was further improved by Hutter et al. (2016) manifested itself in terms of excellent dynamic locomotion. Among these state-of-the-art designs, whole-body modeling serves as a cornerstone for sophisticated control and estimation.

Legged robots are typically treated as floating-based multi-body systems (MBS). Due to the complexity of the whole-body models, a common pipeline is to adopt reduced-order models to concentrate on some significant degrees of freedom. In Raibert, Brown & Chepponis (1984), the concept of virtual legs was introduced to stimulate a 3D one-legged hopping. By controlling the jumping height, forwarding speed, and body pose of the machine, a successfully balanced hopping was achieved in the 3D environment. The idea was further extended to a four-legged robot shown in Raibert, Chepponis & Brown (1986). The linear inverse pendulum (LIP) model, on the other hand, is one of the most widely used template models for both biped and quadruped robots (Kajita et al., 2003). The LIP model mainly relies on the zero-moment point (ZMP) concept, which roughly acts as the center of pressure concerning all ground reaction forces. Besides, the spring-loaded inverted pendulum (SLIP) model was adopted to mimic the spring-like behavior of a robotic leg in the running motion (Hutter et al., 2010). In addition to the LIP and SLIP models, the centroidal momentum model further studies the effect of angular momentum on the body, and it was used to generate a force/position hybrid strategy that allowing the HyQ robot to stand and walk on slopes above 50° (Focchi et al., 2017).

The template model alone, in general, cannot enable dynamic-legged locomotion, and a precise whole-body dynamics model is required. Based on whole-body dynamics models, advanced control scheme such as model predictive control (MPC) has been applied to real-world legged robots. In Bellicoso et al. (2017), the authors designed the quadruped ANYmal, which is actuated by series-elastic actuators. By using a hierarchical whole-body controller relied on ZMP to optimize the whole-body motion and contact forces to execute dynamic gaits, including trot, pace, and dynamic lateral walk, as well as a smooth transition between them. The quadruped cheetah developed by MIT also used a full-dynamic parameterized model (Bledt et al., 2018). By assembling an actuator for high force proprioceptive control, the quadruped can climb stairs without any sensor. To improve the stability performance of a humanoid robot, the authors in Xie, Zhao & Mei (2015) applied a whole-body control scheme based on the relative position of feet and the trajectory of its CoM with a ZMP regulation. In Xin et al. (2019), a whole-body dynamic model is developed, which consists of a dynamic torso model, a dynamic wheel-leg model, and contact force constraints between the wheels and the ground. Based on the whole-body dynamic model, they proposed a control frame to generate whole-body motions on a wheel-leg robot for dynamic locomotion and balance.

The main obstacle of applying the whole-body dynamic model, considering all parts’ inertias, is the complication of the dynamic model. A quadruped robot typically needs fast response performance with high frequency during locomotion. In practice, a tractable whole-body dynamic model is critical since it only costs a few moments for a legged robot to take a step, and a feasible solution may not be available in the model is too complicated. Screw theory is an advanced robot modeling method, which is generally used for kinematics and dynamics modeling of robots (Cibicik & Egeland, 2019; De Jong, Van Dijk & Herder, 2019; Frisoli et al., 2011; Gallardo-Alvarado, Rodrguez-Castro & Delossantos-Lara, 2018; Du, Fnadi & Benamar, 2020), especially for those involving parallel mechanisms. One advantage of the screw theory is that it can simplify the robot coordinate system and make the solution faster. In light of the screw theory, the authors in Chen et al. (2015) proposed the fault-tolerant gait to deal with the kinematics problem containing mechanical faults.

In this paper, a whole-body dynamic modeling method for quadruped robots is proposed, which can make a quadruped robot realize real-time motions of a loop in 1 ms and generate more stable movements.

This paper mainly contributed as follows:

1. A novel modeling method for quadruped robots is proposed, and based on screw theory, both kinematic and dynamic models can be formulated elegantly.
2. A model-based control strategy is proposed, which can improve the dynamic response performance of the robot.
3. We integrate the proposed model and plan on the “XiLing” robot, which has high dynamic response performance in a complex environment. Various simulations and experiments are carried out to validate the method’s effectiveness.

## System Overview

**Figure 1:**
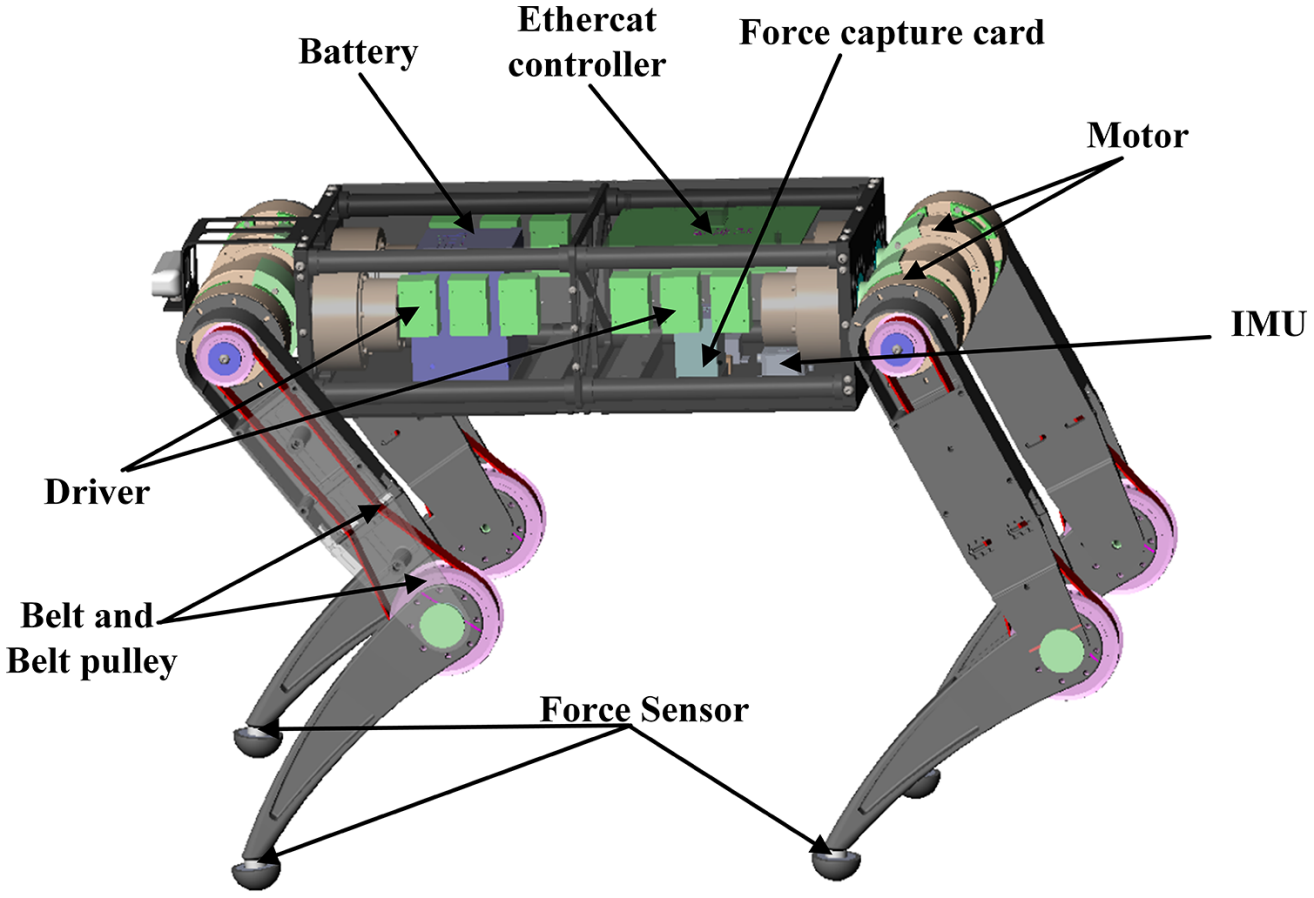
Main components of “XiLing”. The “XiLing” robot is an electrically powered agility robotcapable of locomotion in complex environments.

This paper introduces a new modeling method for quadruped robots based on screw theory to improve the dynamic performance in a complex ground environment. Our design, “XiLing” is shown in Fig. 1. To increase the carrying capacity of the robot, we use carbon fiber and aluminum alloy to reduce the overall weight without losing strength.

As shown in Fig. 1, each leg has three degrees of freedom. The abduction/adduction joint contributes to the leg motion in the frontal plane. The hip and knee joint commonly relate to the leg motion in the sagittal plane. The shank uses a pulley to drive, which the reduction ratio is 1:2. The robot can move in all directions with a walk or trot gait. The whole-body dynamics model is built to analyze the dynamics characteristics during walking. In addition, we design and assemble the motor drive module and add the brake system to ensure the safety of the robot itself and the operator.

**Figure 2:**
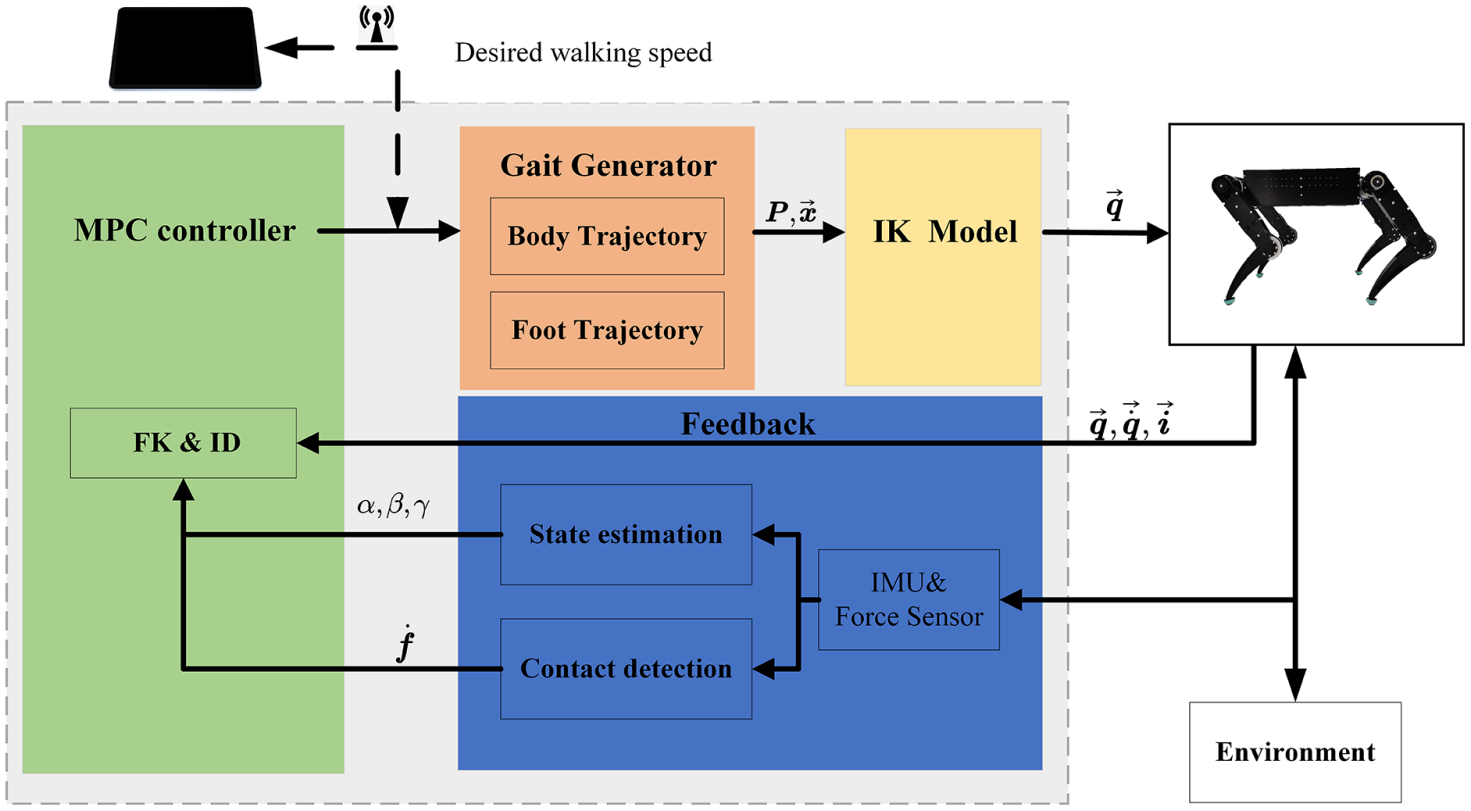
Quadruped robot control framework. The user sends gait type and speed commands to the Industrial Personal Computer by a webpage. The gait generator creates the trajectory of the body and foot. Then use the IK model to compute the joint parameters. The change of the environment and robot statesis used to make real-time adjustments to the movement trajectory.

Moreover, force sensors are placed on the toes of each leg, which can sense the ground reaction in real-time. Thus, we can analyze the changes in external terrain and the position and posture of the robot body in combination with the IMU. Based on the perception of the external environment and itself, the current state can be comprehensively estimated. The corresponding dynamic model will be selected according to the topological structure at this time. To calculate the torque of the joint at this time. Then the MPC controller will re-plan the walking gait and trajectory so that the robot can walk smoothly. The control framework is shown in Fig. 2.

As we know, the legged robot has to face the extraordinarily complex and changeable external environment in walking, and it needs to respond to the changes of the external environment. Otherwise, the feet may have landed, and the algorithm has not been solved yet; thus, the algorithm has lost its due function, which is also one reason why no one has established the whole-body dynamics model of the quadruped robot at present. We use the screw theory to develop the dynamic quadruped model. It will significantly simplify the establishment of the coordinate system and improve the model calculation speed and efficiency. The following several sections will detail the modeling process of kinematic and inverse dynamic.

## Kinematics

Solving kinematics is the foundation of dynamic calculation. Before solving the dynamics, we need to finish the kinematics to obtain the position and velocity of each link. This section will introduce the definition of the coordinate system, inverse kinematic, and forward kinematic. It will provide the theoretical basis for subsequent modeling and planning.

### Definition of coordinate system

**Figure 3:**
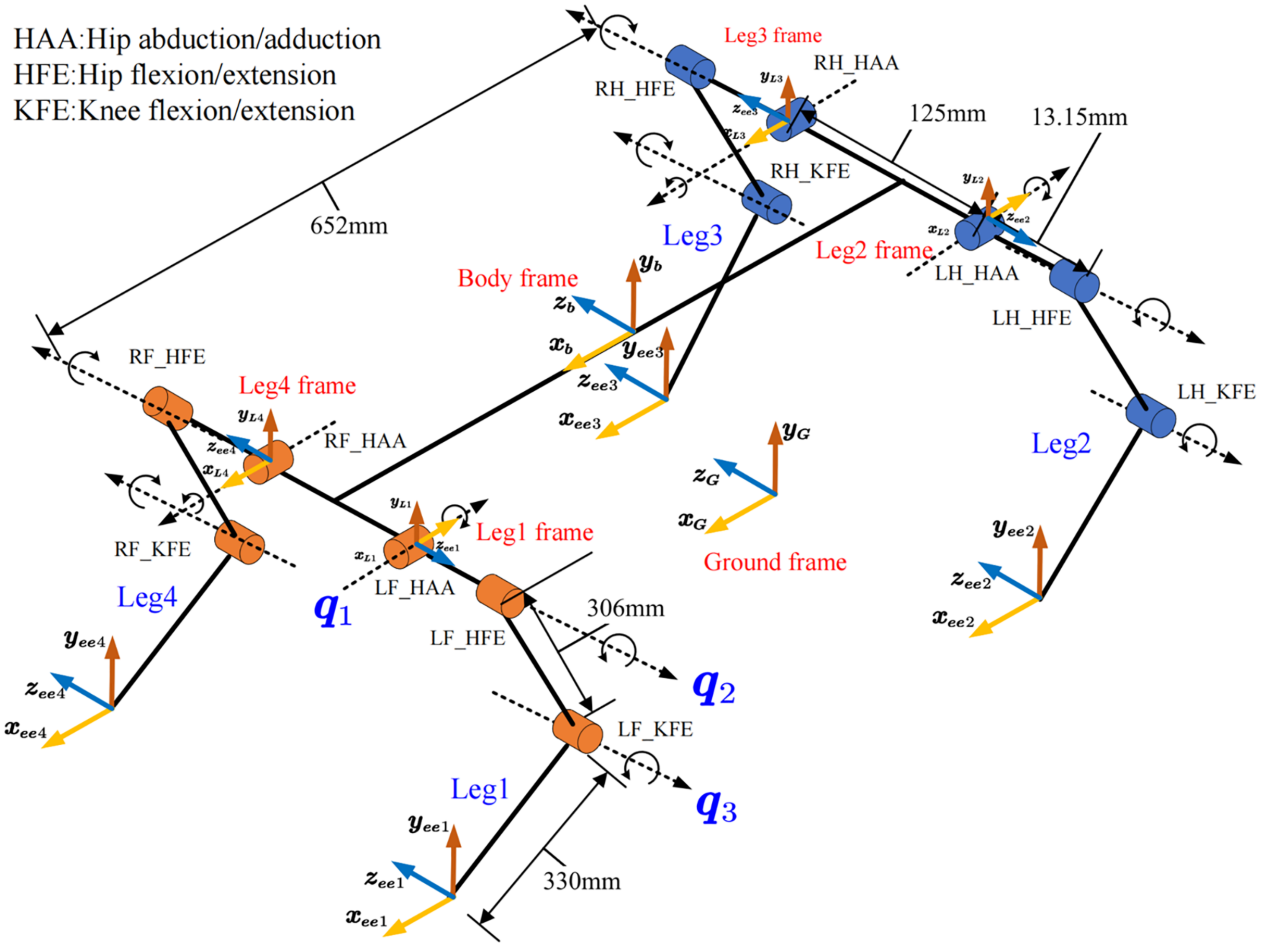
Home position and coordinate definition of robot. The frame of body, leg and foot are defined and the position and posture are illustrated.

The link’s rotation axis, velocity, and inertia are expressed differently in different coordinates. So, it is essential to define a unified coordinate system. Figure 3 shows the definition of the home position and the coordinate of body center, leg, and ground. The leg frame is defined at the intersection of the joint HAA (hip abduction/adduction) and HFE (Hip flexion/extension) axes in the same direction as the body frame.

### Inverse kinematic

The inverse kinematics is given based on the leg frame’s representation $\vec x$ in the leg frame to calculate the joint rotation angle $\vec q$. Figure 3 shows the coordinates and steering definitions for each joint of the leg. Before solving the problem, we simplify the calculation by limiting the actual walking condition of the robot. Assume that the robot’s toes will not reach the top of the body, that is:

(1) $$\left| {{{\it q}_2}} \right| < \displaystyle{\pi \over 2}{\kern 1pt} {\kern 1pt} \Leftrightarrow {\kern 1pt} {{\kern 1pt} ^L}y < 0$$

First, *q*_1_ is calculated by projecting onto the *YOZ* plane. In the *YOZ* plane, the projection of point *C* is *D*. We can obtain Eq. (2) by considering the geometrical relationship:

(2) $$\alpha = {\rm arc}\cos \displaystyle{{\left| {z} \right|} \over {\sqrt {{{ y}^2} + {{z}^2}} }},\quad \quad \beta = {\rm arc}\cos \displaystyle{{{{ l}_1}} \over {\sqrt {{{ y}^2} + {{z}^2}} }}$$

Here *α* is the angle between $\overrightarrow {{DO}}$ and *Z*, and *β* is the angle between $\overrightarrow {{AO}}$ and $\overrightarrow {{DO}}$.

**Figure 4:**
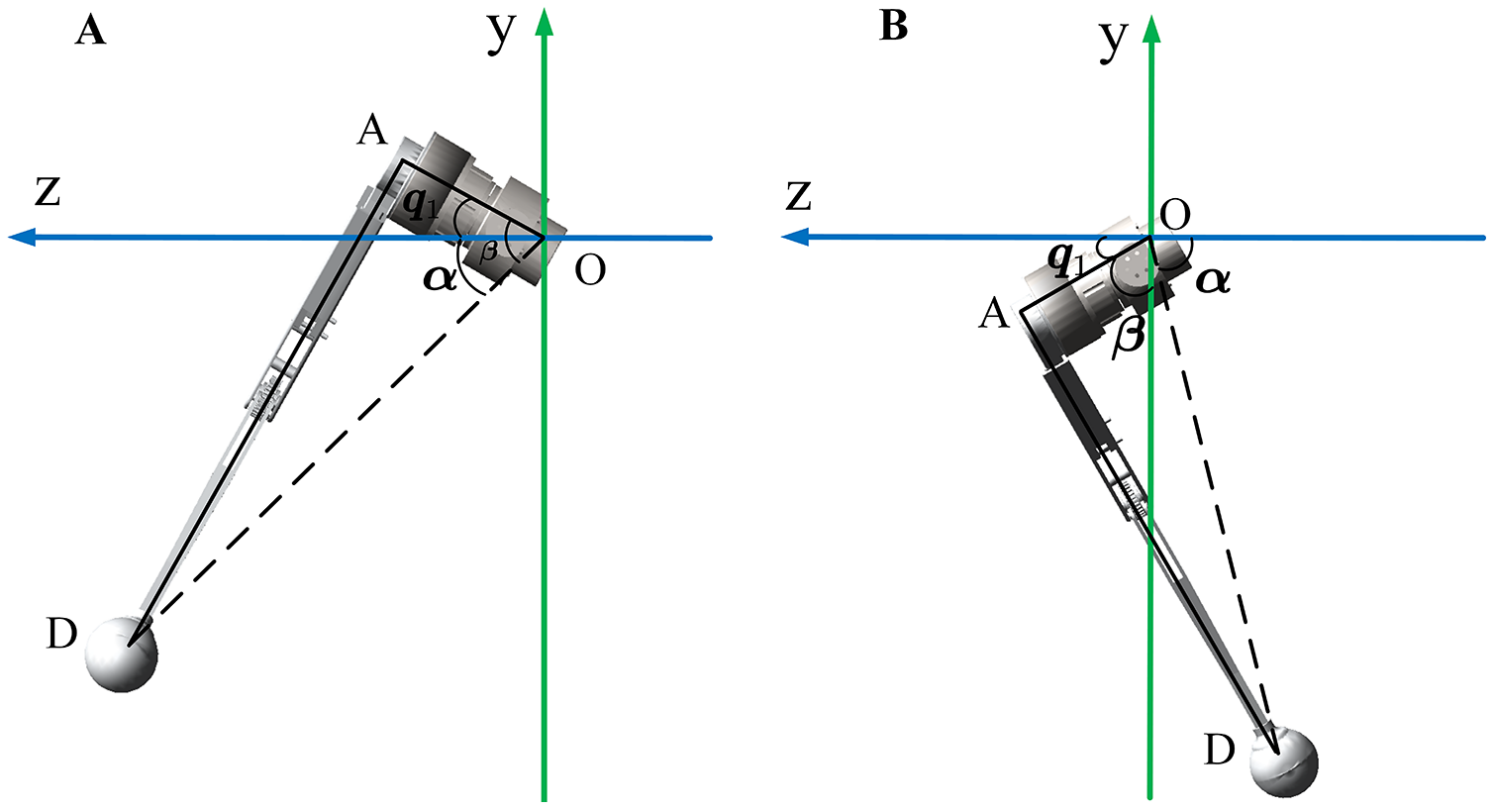
Geometric relations of a single leg in the YOZ plane. The leg end-effector position has twodifferent cases when robot walking. Both of them have different solvers.

Figure 4 shows the geometric relationship of the ends under different positions. We can use this to calculate *q*_1_ according to Eq. (3).

(3) $${{q}_1} = \left\{{\matrix{{\hskip-1.5pc}{\alpha - \beta }  {{y} \lt 0,{z} \gt 0} \\ {\pi - \alpha - \beta }  {{y} \lt 0,{z} \lt 0} \cr }} \right.$$

**Figure 5:**
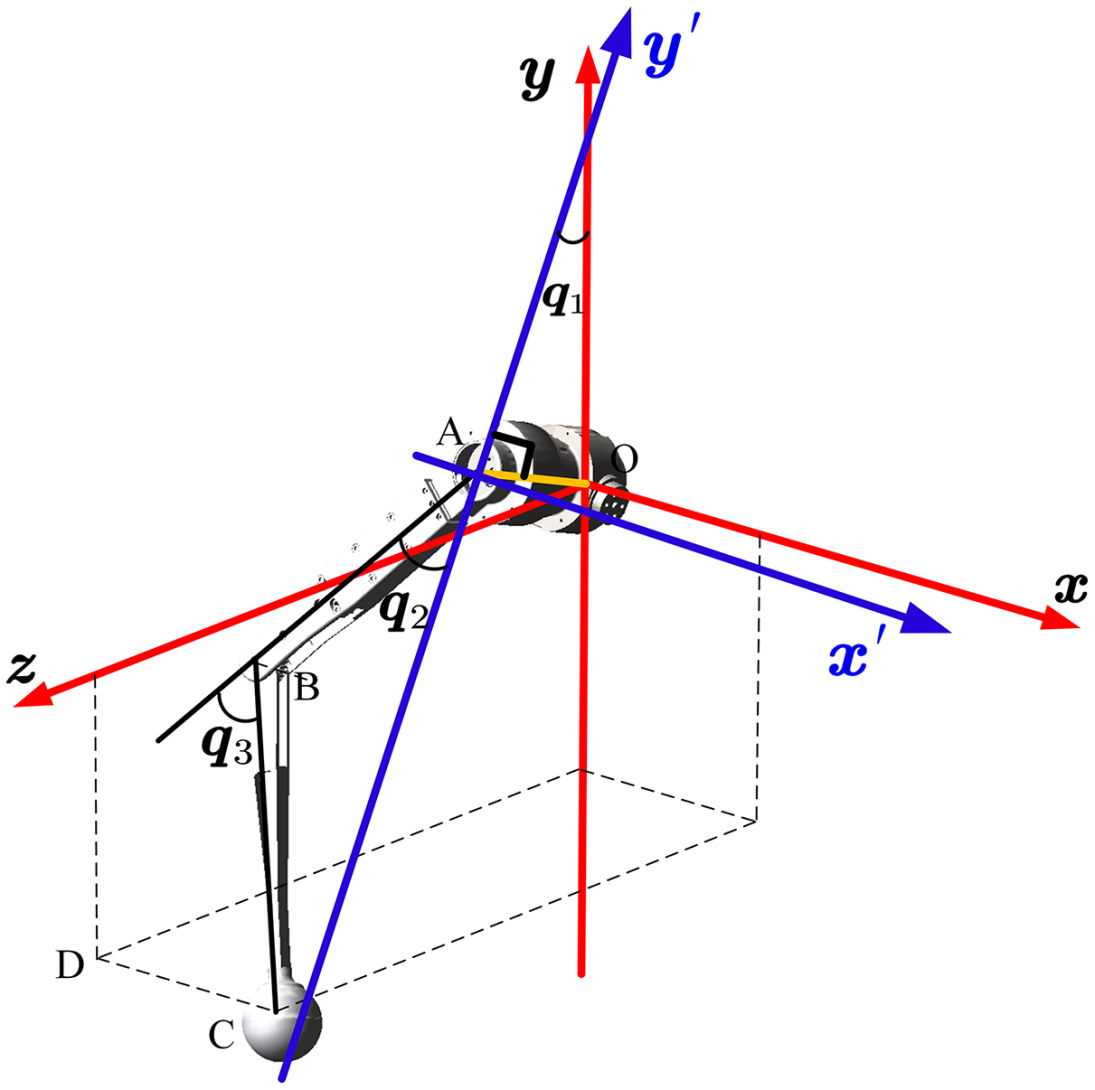
The illustrated conversion between the old and new frame of the leg.

During the robot locomotion, the upper and lower links are always in the same plane. This plane is used as a new study plane to calculate *q*_2_ and *q*_3_. The new origin of the coordinate system was transferred to joint HFE, as shown in Fig. 5.

The conversion formula of the old and new coordinate systems is Eq. (4):

(4) $$\matrix{{\hskip-7.8pc}{{{x}^{\prime}} = {x}} \\ {{y}^\prime = - {AD} = - \sqrt {{{y}^2} + {{z}^2} - {l}_1^2} }}$$

Then the problem was transformed into solving the inverse kinematics of the planar two-link mechanism. According to the trigonometric relationship, we know that:

(5) $${\phi} = {\rm arc}\cos \displaystyle{{\left| {{x}\prime} \right|} \over {\sqrt {{x}{\prime^2} + {y}{\prime^2}} }},\quad \phiv = {\rm arc}\cos \displaystyle{{{l}_2^2 + {x}{\prime^2} + {y}{\prime^2} - {l}_3^2} \over {2{{l}_2}\sqrt {{x}{\prime^2} + {y}{\prime^2}} }}$$

**Figure 6:**
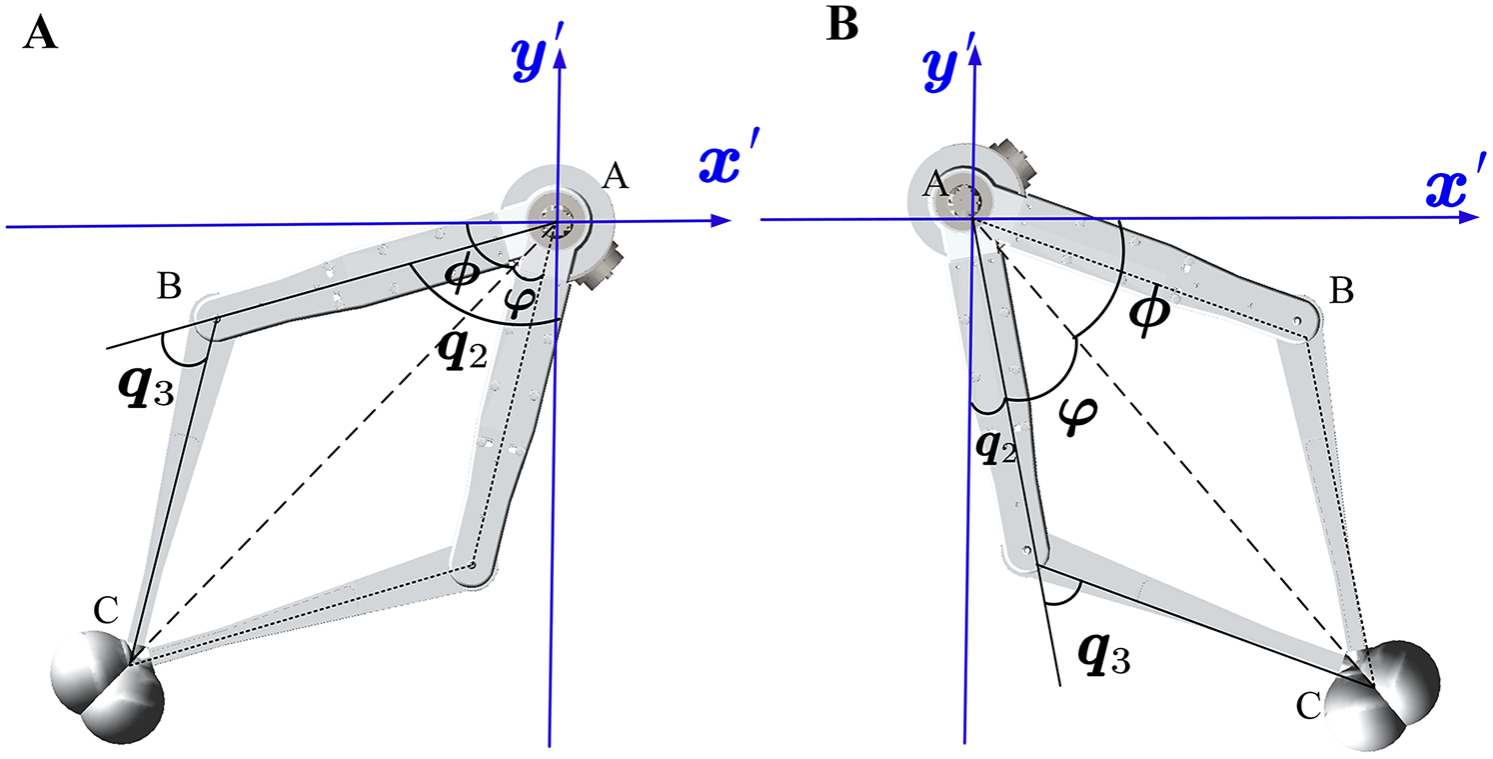
The relationship between the position of the link and angle of the joint in the new leg frame. The foot position has two cases and each case has two solutions. It is chosen according to the walking states.

Here *ϕ* is the angle between $\overrightarrow {{AC}}$ and the *x*-axis and *φ* is the angle between $\overrightarrow {{AC}}$ and $\overrightarrow {{AB}}$. As shown in Fig. 6, when the end effectors are in a specific position, there will be different solutions due to different ways to bend the legs.

(6) $$\matrix{{\hskip-20.6pc}{{if}\quad {{q}_3} \gt 0} \\ {\!\!\!\!\!\!{{q}_2} = \left\{ {\matrix{ {\displaystyle{\pi \over 2} -  - \phi }  {{{x}^{\prime}} \gt 0,{y}\prime \lt 0} \cr { - \displaystyle{\pi \over 2} -  + \phi }  {{{x}^{\prime}} \lt 0,{y}\prime \lt 0} \\} } \right. \quad {{q}_3} = {\rm arc}\cos \displaystyle{{{l}_2^2 + {l}_3^2 - {x}{\prime^2} - {y}{\prime^2}} \over {2{{l}_2}{{l}_3}}}}\\ {{\hskip-20.6pc}{if}\quad {{q}_3} \lt 0} \\ {{{q}_2} = \left\{ {\matrix{ {\displaystyle{\pi \over 2} +  - \phi }  {{{x}^{\prime}} > 0,{y}\prime < 0} \\ { - \displaystyle{\pi \over 2} +  + \phi }  {{{x}^{\prime}} < 0,{y}\prime < 0} \\} } \right.\quad {{q}_3} = - {\rm arc}\cos \displaystyle{{{l}_2^2 + {l}_3^2 - {x}{\prime^2} - {y}{\prime^2}} \over {2{{l}_2}{{l}_3}}}} \\ }$$

### Forward kinematic

Forward kinematics is to calculate the expression of all links relative to the ground frame given the initial position and joint rotation angle. Because the robot is a floating base system, we cannot directly find the expression of the relevant parameters of the robot in the ground frame. So we first take the center of the robot body as the reference frame to solve the presentation of all links. And then, we transfer them to the ground coordinate according to the conversion formula of the body and the ground coordinate system. Screw theory is used to build a kinematics model. See Appendix A for the meaning of all symbols used in this paper.

In the screw theory, the velocity screw *V* and screw axis *S* of the rigid body is defined by a pair of vectors that is:

(7) $$S = \left( {{T};{R}} \right)\quad V = \left( {{v},{w}} \right)$$

The screw axis *S* defines the position and positive rotation direction of the revolute joint. The first vector is the position on the ground coordinate frame, and the second vector is the direction of rotation. *V* represents the velocity and angular velocity of the rigid body.

In this paper, all the joint and link parameters are transferred to the body frame *B* for analysis. So, we assume that the body coordinate frame is relatively stationary when we solve the forward kinematic. The forward position model solves the end-effector position on the frame *B* from the inputs angle of every joint. Before computing the angle, the initial position of the robot should be determined to determine the initial velocity screw as follows:

(8) $${\hskip-1pc}\matrix{ {} \hfill  {^B{S_{11}} = \left( {{B_l}/2,0, - {B_w}/2, - 1,0,0} \right)} \hfill \cr {} \hfill  {^B{S_{12}} = \left( {{B_l}/2,0, - {B_w}/2 - {l_1},0,0, - 1} \right)} \hfill \cr {} \hfill  {^B{S_{13}} = \left( {{B_l}/2, - {l_2}, - {B_w}/2 - {l_1},0,0, - 1} \right)} \hfill \cr {} \hfill  {^B{S_{21}} = \left( { - {B_l}/2,0, - {B_w}/2, - 1,0,0} \right);} \hfill \cr {} \hfill  {^B{S_{22}} = \left( { - {B_l}/2,0, - {B_w}/2 - {l_1},0,0, - 1} \right);} \hfill \cr {} \hfill  {^B{S_{23}} = \left( { - {B_l}/2, - {l_2}, - {B_w}/2 - {l_1},0,0, - 1} \right)} \hfill \cr {} \hfill  {^B{S_{31}} = \left( { - {B_l}/2,0,{B_w}/2,1,0,0} \right);} \hfill \cr {} \hfill  {^B{S_{32}} = \left( { - {B_l}/2,0,{B_w}/2 + {l_1},0,0,1} \right);} \hfill \cr {} \hfill  {^B{S_{33}} = \left( { - {B_l}/2, - {l_2},{B_w}/2 + {l_1},0,0,1} \right)} \hfill \cr {} \hfill  {^B{S_{41}} = \left( {{B_l}/2,0,{B_w}/2,1,0,0} \right);} \hfill \cr {} \hfill  {^B{S_{42}} = \left( {{B_l}/2,0,{B_w}/2 + {l_1},0,0,1} \right);} \hfill \cr {} \hfill  {^B{S_{43}} = \left( {{B_l}/2, - {l_2},{B_w}/2 + {l_1},0,0,1} \right)} \hfill \cr }$$

We can obtain the initial velocity screw of the joint computed according to the screw axis *S*_***ij***_ using Eq. (9). Where $\hat {\bf s}$ is the unit velocity screw. Such as the joint HAA of leg1 is $\hat {\bf s} = \left( {0,0,0,1,0,0} \right)$.

(9) $$J_{vso}^{ij} = {T_v}\left( {{S_{ij}}} \right) \cdot \hat s$$

Then we can get the end-effector position as Eq. (10) and the Jacobian matrix in Eq. (11).

(10) $$e{e_i} = P\left( {J_{vso}^{i1} \cdot {\theta_{i1}}} \right)P\left( {J_{vso}^{i2} \cdot {\theta_{i2}}} \right)P\left( {J_{vso}^{i3} \cdot {\theta_{i3}}} \right)e{e_{io}}$$

(11) $${J_i} = \left[T_v\left( P_0 \right)J_{vso}^{1i}\quad T_v\left( P_{1i} \right)J_{vso}^{2i}\quad {T_v}\left( P_{2i} \right)J_{vso}^{3i}\right]$$

where *i* = 1, 2, 3, 4 is the leg number of the robot; *P* is the homogeneous transformation matrix when the joint rotation *θ*. *S*_*ij*_ is the initial velocity screw of the joint computed according to the screw axis in home position.

## Inverse Dynamics

Dynamics is to solve the joint torque when the current motion state and external force are known. The position and velocity of all the links and joints are required before the dynamics. The previous section addresses these issues. Additionally, the inertia and the constraint matrix are also known because these two parameters are only related to the robot’s position.

In this paper, all the joints are revolute joints. Each joint has five dimensions of constraint and one dimension of motion. Therefore, we can define the constraint matrix and moment matrix according to the Plucker basis coordinate system. At the home position, it is expressed as Eq. (12).

(12) $$^GJ_{cmo}^{ij} = {T_f}\left( {{S_{ij}}} \right) \cdot {\hat s_{6 \times 5}},{\quad ^G}M_{cmo}^{ij} = {T_f}\left( {{S_{ij}}} \right) \cdot {\hat s_{6 \times 1}}$$

At any given time, the constraint matrix depends only on the position of the joint. According to the forward kinematics, the homogeneous transformation matrix can be obtained according to Eq. (10). And then the constraint matrix at any time can be obtained by Eq. (13):

(13) $$^GJ_{cm}^{ij} = {T_f}{\left( P \right)^G}M_{cmo}^{ij},{\quad ^G}M_{cm}^{ij} = {T_f}{\left( P \right)^G}M_{cmo}^{ij}$$

For the inertia of the linkage, we can first get the inertia of the linkage at the center of mass. Then, according to the position relationship between the center of mass and the ground, Eq. (14) is used to solve it.

(14) $$^G{I_{ij}} = {^G}{T_f}\left( {{P_{ij}}} \right){I_{ijo}}^GT_f^T\left( {{P_{ij}}} \right)$$

where *T*_*f*_ and *P* are isomorphic and only depends on the position of the links. *I*_*ijo*_ is the initial inertia details in Appendix B. Moreover, we can combine ^*G*^*I*_*ij*_ to a big matrix: $I = {\rm diag}\left( {^G{I_{ij}}} \right)$.

**Figure 7:**
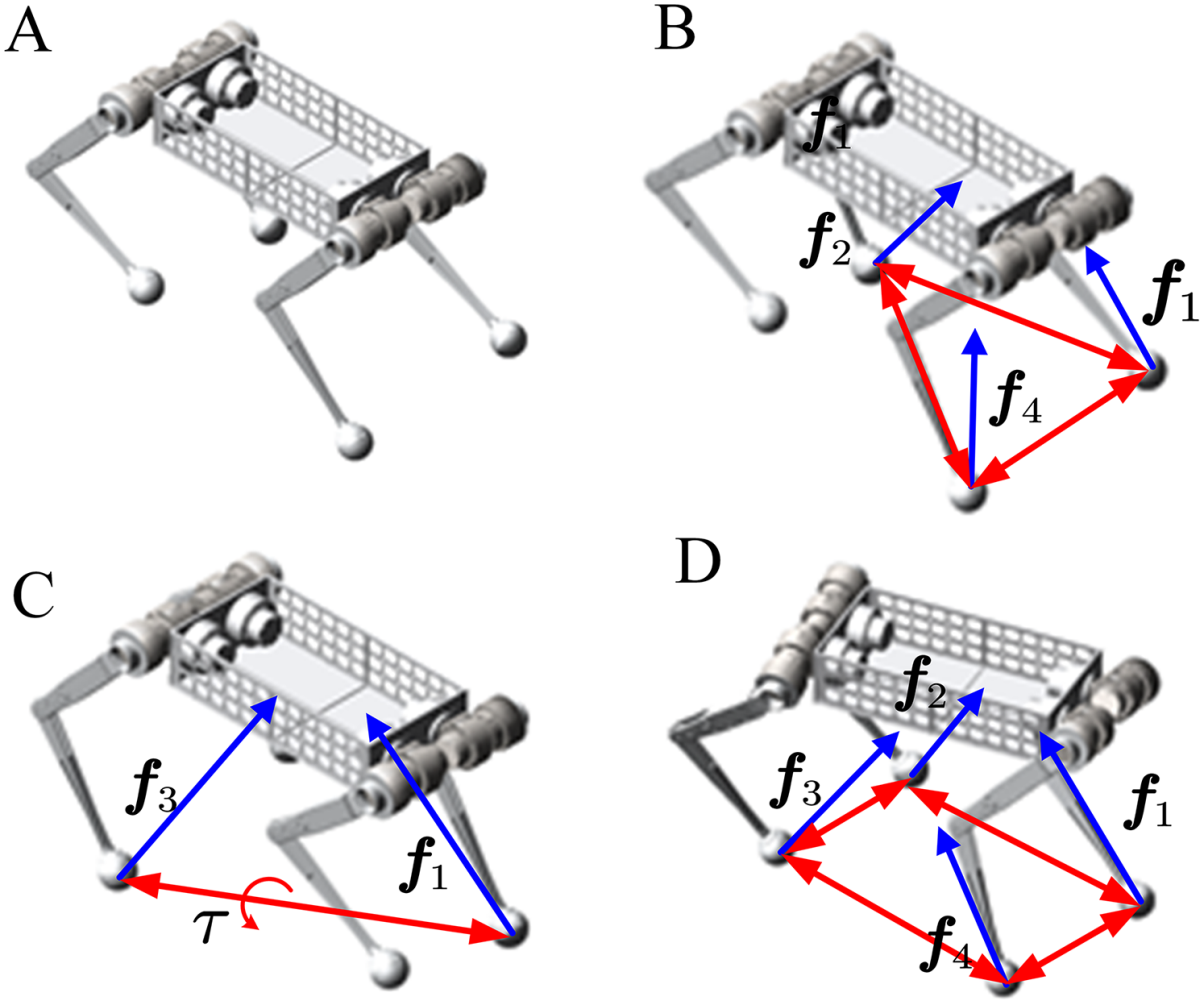
Topological structure of dynamic model under different states. The blue arrow indicatesthe ground reaction which is the foot in contact with the ground. The red arrows surround supporting polygons. (A) Floating phase: running in bound gait. (B and C) Walking phase: running in walk or trotgait. (D) Standing phase: running in the stand which can pitch, roll, yaw.

The solution of velocity is related to the model of dynamics. During the walking phase, quadruped robots mainly have three situations: four-leg landing on the ground, two or three landing on the ground, and four suspended in the air. Respectively corresponding to the stand, walk and bound. In this paper, different states of robots are divided into different topological for analysis. Furthermore, the dynamics model is different under different topological mechanisms. In the state of stand and walk, the robot is a fixed base system, and the velocity of all the links can be obtained by establishing a constraint matrix. Then it can be substituted into the dynamic model to calculate the joint torque; in the bound gaits, the robot is a floating base system, which the constraint matrix is singular. Here, we first get the acceleration according to the last time velocity, then use the integral method to get the next velocity. As shown in Fig. 7, the topology is constructed for these three cases. Figure 7A shows that the robot jumps and is free from the ground reaction. Figures 7B and 7C show the external forces on the robot when it walks in the trot or walking gaits. Figure 7D shows the force analysis of the robot when it stands, and all four legs are in the support phase. Next, we will introduce how to build the constraint matrix and solve the dynamic model.

### Stand

Show in Fig. 7D, When the robot stand, there is only one end-effector, the body. It can realize the action of squatting and standing up and the three-axis attitude transformation of roll, pitch, and yaw. In this case, each robot’s leg is connected to the ground as a passive spherical joint, and the three-dimensional translation is constrained. The body is the only end effector, and the robot can be regarded as a parallel mechanism. The force of each joint on its parent connecting link is defined as negative, and the force on the child connecting link is defined as positive so that the constraint matrix can be established.

Once we have the constraint matrix for each joint, write the constraint matrix for all joints and links as a larger matrix *C*.

(15) $$C=\matrix{\\ ground \\ body \\ L_{11}\\ L_{12}\\ L_{13}\\ L_{21}\\ L_{22}\\ L_{23}\\ L_{31}\\ L_{32}\\ L_{33}\\ L_{41}\\ L_{42}\\ L_{43}} \left[ \matrix{ fix  s_1  s_2  s_3  s_4  r_{11}  r_{12}  r_{13}  r_{21}  r_{22}  r_{23}  r_{31}  r_{32}  r_{33}  r_{41}  r_{42}  r_{43}  m_{11}  m_{12}  m_{13}  m_{21}  m_{22}  m_{23}  m_{31}  m_{32}  m_{33}  m_{41}  m_{42}  m_{43} \\ 1 & 1 & 1 & 1 & 1 & 0 & 0 & 0 & 0 & 0 & 0 & 0 & 0 & 0 & 0 & 0 & 0 & 0 & 0 & 0 & 0 & 0 & 0 & 0 & 0 & 0 & 0 & 0 & 0 \\ 0 & 0 & 0 & 0 & 0 & -1 & 0 & 0 & -1 & 0 & 0 & -1 & 0 & 0 & -1 & 0 & 0 & -1 & 0 & 0 & -1 & 0 & 0 & -1 & 0 & 0 & -1 & 0 & 0 & \\ 0 & 0 & 0 & 0 & 0 & 1 & -1 & 0 & -1 & 0 & 0 & 0 & 0 & 0 & 0 & 0 & 0 & 1 & -1 & 0 & -1 & 0 & 0 & 0 & 0 & 0 & 0 & 0 & 0 \\ 0 & 0 & 0 & 0 & 0 & 0 & 1 & -1 & 0 & 0 & 0 & 0 & 0 & 0 & 0 & 0 & 0 & 0 & 1 & -1 & 0 & 0 & 0 & 0 & 0 & 0 & 0 & 0 & 0 \\ 0 & -1 & 0 & 0 & 0 & 0 & 0 & 1 & 0 & 0 & 0 & 0 & 0 & 0 & 0 & 0 & 0 & 0 & 0 & 1 & 0 & 0 & 0 & 0 & 0 & 0 & 0 & 0 & 0 \\ 0 & 0 & 0 & 0 & 0 & 0 & 0 & 0 & 1 & -1 & 0 & 0 & 0 & 0 & 0 & 0 & 0 & 0 & 0 & 0 & 1 & -1 & 0 & 0 & 0 & 0 & 0 & 0 & 0 \\ 0 & 0 & 0 & 0 & 0 & 0 & 0 & 0 & 0 & 1 & -1 & 0 & 0 & 0 & 0 & 0 & 0 & 0 & 0 & 0 & 0 & 1 & -1 & 0 & 0 & 0 & 0 & 0 & 0 \\ 0 & 0 & -1 & 0 & 0 & 0 & 0 & 0 & 0 & 0 & 1 & 0 & 0 & 0 & 0 & 0 & 0 & 0 & 0 & 0 & 0 & 0 & 1 & 0 & 0 & 0 & 0 & 0 & 0 \\ 0 & 0 & 0 & 0 & 0 & 0 & 0 & 0 & 0 & 0 & 0 & 1 & -1 & 0 & 0 & 0 & 0 & 0 & 0 & 0 & 0 & 0 & 0 & 1 & -1 & 0 & 0 & 0 & 0 \\ 0 & 0 & 0 & 0 & 0 & 0 & 0 & 0 & 0 & 0 & 0 & 0 & 1 & -1 & 0 & 0 & 0 & 0 & 0 & 0 & 0 & 0 & 0 & 0 & 1 & -1 & 0 & 0 & 0 \\ 0 & 0 & 0 & -1 & 0 & 0 & 0 & 0 & 0 & 0 & 0 & 0 & 0 & 1 & 0 & 0 & 0 & 0 & 0 & 0 & 0 & 0 & 0 & 0 & 0 & 1 & 0 & 0 & 0 \\ 0 & 0 & 0 & 0 & 0 & 0 & 0 & 0 & 0 & 0 & 0 & 0 & 0 & 0 & 1 & -1 & 0 & 0 & 0 & 0 & 0 & 0 & 0 & 0 & 0 & 0 & 1 & -1 & 0 \\ 0 & 0 & 0 & 0 & 0 & 0 & 0 & 0 & 0 & 0 & 0 & 0 & 0 & 0 & 0 & 1 & -1 & 0 & 0 & 0 & 0 & 0 & 0 & 0 & 0 & 0 & 0 & 1 & -1 \\ 0 & 0 & 0 & 0 & -1 & 0 & 0 & 0 & 0 & 0 & 0 & 0 & 0 & 0 & 0 & 0 & 1 & 0 & 0 & 0 & 0 & 0 & 0 & 0 & 0 & 0 & 0 & 0 & 1 } \right] $$

According to the formula of constraint matrix, *C*_*ij*_ represents the force of joint *j* on link *i*. For constraint: ${C_{ij}} = {^G}J_{cmo}^{ij}$; For motion: ${C_{ij}} = {^G}M_{cmo}^{ij}$.

Each of these rows is a linkage, there are 14 of them, and each of these columns is a joint. For the same joint, constraints and motions are considered separately. We knew that *C* is a sparse matrix, and we can use unique algorithms to calculate it to improve the computational efficiency of dynamics. We know that by conservation of energy in Eq. (16).

(16) $${C^T} \cdot v = {C_v} = \left( {\matrix{ 0 \\ {\dot \theta}} } \right)$$

where, *C*_*v*_ represents the power of joint rotation, and *v* represents the speed of all the links. Take the derivative of both sides:

(17) $${C^T} \cdot \dot v = \left( {\matrix{ 0 \\ {\ddot \theta} } } \right) - {\dot C^T} \cdot v$$

Define: ${C_a} = \left( {\matrix{ 0 \cr {\ddot \theta } \cr } } \right) - {\dot C^T} \cdot v$ , then *C*^*T*^*a* = *C*_*a*_

For any linkage, the force equilibrium condition satisfies Eq. (18):

(18) $${f_p} = - Ia + {f_c} = - {f_e} - Ig + v{ \times ^*}Iv$$

By combining the equilibrium equations of all the links and Eq. (17), we can take the dynamic Eq. (19) of the whole-body dynamic:

(19) $$\left[ {\matrix{ { - I}  C \\ {{C^T}}  {} \\} } \right]\left[ {\matrix{ a \\ \eta \\ } } \right] = \left[ {\matrix{ {{f_p}} \\ {{c_a}} \\ } } \right]$$

Here *a* is the acceleration of all links and *η* is the forces of all joints, including constraint and driving force.

### Walk

Quadruped robots can walk in various gaits, such as walk, trot, pace, gallop. The main differences between gaits are the order and the time of the stride and the duty cycle of the swing phase. Regardless of the gait, there always exists a supporting phase and a swinging phase at any given moment. The dynamic model was constructed with the contact point between the support and ground as a spherical joint and the swinging leg and body as the end-effectors. This section introduces how to build a constraint matrix by taking Walk-gait as an example. The methods are the same for other gaits. We need to analyze which leg is in the swing and which portion supports and modifies the constraints.

When the robot walks in Walk-gait, at any time, there are three legs in contact with the ground and one leg in the air. There are four different topologies. Figure 7B shows the third leg in the air. This section analyzes only this case, and other issues are similar. Among them, the 1st, 2nd, and 4th legs contribute to the support contact with the ground and support body movement. The contact points between the toe and the ground can be regarded as spherical joints, which constraining three-dimensional translation, but three-dimensional rotation is not restricted. Therefore, the constraint of a spherical joint can be obtained as follows:

${s_i} = \left[ {\matrix{ 1  0  0 \cr 0  1  0 \cr 0  0  1 \cr 0  0  0 \cr 0  0  0 \cr 0  0  0 \cr } } \right]$

Furthermore, the constraint matrices of all the joints can be combined into a larger matrix *C* to represent the robot’s force. The establishment and solution of the dynamic model are consistent with the above Stand topology, which will not be described in detail.

(20) $${\hskip-17.3pc}C= \matrix{ \\ ground \\ body \\ L_{11}\\ L_{12}\\ L_{13}\\ L_{21}\\ L_{22}\\ L_{23}\\ L_{31}\\ L_{32}\\ L_{33}\\ L_{41}\\ L_{42}\\ L_{43}\\ } \left[ \matrix{ fix  s_1  s_3  r_{11}  r_{12}  r_{13}  r_{21}  r_{22}  r_{23}  r_{31}  r_{32}  r_{33}  r_{41}  r_{42}  r_{43}  m_{11}  m_{12}  m_{13}  m_{21}  m_{22}  m_{23}  m_{31}  m_{32}  m_{33}  m_{41}  m_{42}  m_{43} \\ 1  1  1  0  0  0  0  0  0  0  0  0  0  0  0  0  0  0  0  0  0  0  0  0  0  0  0 \\ 0  0  0  -1  0  0  -1  0  0  -1  0  0  -1  0  0  -1  0  0  -1  0  0  -1  0  0  -1  0  0 \\ 0  0  0  1  -1  0  -1  0  0  0  0  0  0  0  0  1  -1  0  -1  0  0  0  0  0  0  0  0 \\ 0  0  0  0  1  -1  0  0  0  0  0  0  0  0  0  0  1  -1  0  0  0  0  0  0  0  0  0 \\ 0  -1  0  0  0  1  0  0  0  0  0  0  0  0  0  0  0  1  0  0  0  0  0  0  0  0  0  \\ 0  0  0  0  0  0  1  -1  0  0  0  0  0  0  0  0  0  0  1  -1  0  0  0  0  0  0  0 \\0  0  0  0  0  0  0  1  -1  0  0  0  0  0  0  0  0  0  0  1  -1  0  0  0  0  0  0 \\ 0  0  0  0  0  0  0  0  1  0  0  0  0  0  0  0  0  0  0  0  1  0  0  0  0  0  0 \\ 0  0  0  0  0  0  0  0  0  1  -1  0  0  0  0  0  0  0  0  0  0  1  -1  0  0  0  0 \\ 0  0  0  0  0  0  0  0  0  0  1  -1  0  0  0  0  0  0  0  0  0  0  1  -1  0  0  0 \\ 0  0  -1  0  0  0  0  0  0  0  0  1  0  0  0  0  0  0  0  0  0  0  0  1  0  0  0 \\ 0  0  0  0  0  0  0  0  0  0  0  0  1  -1  0  0  0  0  0  0  0  0  0  0  1  -1  0 \\ 0  0  0  0  0  0  0  0  0  0  0  0  0  1  -1  0  0  0  0  0  0  0  0  0  0  1  -1 \\ 0  0  0  0  0  0  0  0  0  0  0  0  0  0  1  0  0  0  0  0  0  0  0  0  0  0  1 \\ } \right]$$

### Bound

The Bound-gait differs from the above two. It is a floating base system, including five end effectors. The robot belongs to an unconstrained mechanism at this moment. Note that, the constraint matrix corresponding to the force of the robot is a singular matrix. That makes it impossible to calculate the velocities of all the links by calculating the constraint matrix.

(21) $${\hskip-15.4pc}C=\matrix{ \\ body \\ L_{11}\\ L_{12}\\ L_{13}\\ L_{21}\\ L_{22}\\ L_{23}\\ L_{31}\\ L_{32}\\ L_{33}\\ L_{41}\\ L_{42}\\ L_{43}\\ } \left[ \matrix{ r_{11}  r_{12}  r_{13}  r_{21}  r_{22}  r_{23}  r_{31}  r_{32}  r_{33}  r_{41}  r_{42}  r_{43}  m_{11}  m_{12}  m_{13}  m_{21}  m_{22}  m_{23}  m_{31}  m_{32}  m_{33}  m_{41}  m_{42}  m_{43} \\-1  0  0  -1  0  0  -1  0  0  -1  0  0  -1  0  0  -1  0  0  -1  0  0  -1  0  0 \\ 1   -1  0  -1  0  0  0  0  0  0  0  0  1   -1  0  -1  0  0  0  0  0  0  0  0 \\ 0  1   -1  0  0  0  0  0  0  0  0  0  0  1   -1  0  0  0  0  0  0  0  0  0 \\ 0  0  1  0  0  0  0  0  0  0  0  0  0  0  1  0  0  0  0  0  0  0  0  0 \\ 0  0  0  1   -1  0  0  0  0  0  0  0  0  0  0  1   -1  0  0  0  0  0  0  0 \\ 0  0  0  0  1   -1  0  0  0  0  0  0  0  0  0  0  1   -1  0  0  0  0  0  0 \\ 0  0  0  0  0  1  0  0  0  0  0  0  0  0  0  0  0  1  0  0  0  0  0  0 \\ 0  0  0  0  0  0  1   -1  0  0  0  0  0  0  0  0  0  0  1   -1  0  0  0  0 \\ 0  0  0  0  0  0  0  1   -1  0  0  0  0  0  0  0  0  0  0  1   -1  0  0  0 \\ 0  0  0  0  0  0  0  0  1  0  0  0  0  0  0  0  0  0  0  0  1  0  0  0 \\ 0  0  0  0  0  0  0  0  0  1   -1  0  0  0  0  0  0  0  0  0  0  1   -1  0 \\ 0  0  0  0  0  0  0  0  0  0  1   -1  0  0  0  0  0  0  0  0  0  0  1   -1 \\0  0  0  0  0  0  0  0  0  0  0  1  0  0  0  0  0  0  0  0  0  0  0  1 \\ } \right]$$

Here we are going to use integrals to calculate. Then, when the robot starts to move, its position is known, and its velocity is zero. Then, we can establish constraint matrix Eq. (21) to solve the spatial accelerate.

And then, in the next loop, we can obtain the spatial velocity by numerical integration. Although the acceleration of the link changes from time to time, we use high-frequency real-time control, which can be calculated at the frequency of 1,000 Hz. The speed can be refreshed quickly according to the change of the link acceleration. The control effect has been achieved continuously and steadily.

## Verification of Kinematic and Dynamic Models

The above section introduces the whole body kinematic and dynamic models of a quadruped robot. In our control framework, the kinematics model will be used to transform the trajectory of end-effectors from Cartesian space to axial space. Dynamic models will be used for control and planning, making the robot regenerate the control parameters of the next real-time cycle according to the feedback information. This section will introduce how to generate the trajectory in walk and trot gait, and verify the models in simulation and experiment.

### Motion plan

**Figure 8:**
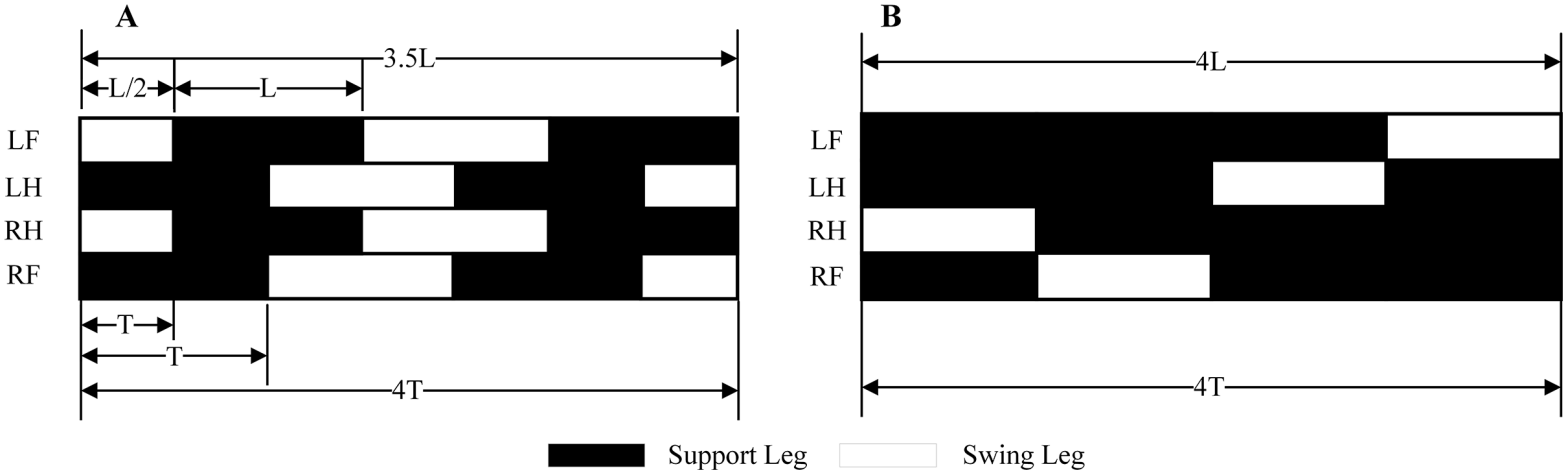
Walk and Trot gaits. The sequence, step length, and time of the robot under walk and trot gaitare described. It will travel half a step-less at the trot gait at the same time. (A) Trot gait. (B) Walk gait.

According to bionics research from Polet & Bertram (2019), for tetrapod, there are different gaits at different speeds. The walking gait is used when motion at a slow pace, using the trot gait when moving faster. Furthermore, use the bound gait when chasing prey or running away. The main difference between different gaits is the order of the stride and the time cycle of the support and swing legs. This paper has mainly introduced the planning of the walk and trot gaits at a slow speed to verify the correctness and feasibility of the model. Figure 8 shows the stride order, stride length, and time to duty ratio in these two gaits. An elliptical trajectory transition is used between two steps, where step length, step height, and step time are adjustable. During the acceleration and deceleration, the displacement of the body is as half as the length of other times. So, we set the step length at the beginning and the end to be half a step, but the duration is the same.

To make the robot walk smoothly and stably, the trajectory needs to satisfy certain constraints, such as the position and velocity should be continuously differentiable. For this purpose, the planning uses the trapezoidal curve, making each step of walking experience a process of acceleration, uniform speed, and deceleration. Equation (22) is the functional expression of the trapezoidal curve, and all following trajectories are planned on this basis. In addition, different curves can be generated by setting different velocity and acceleration.

(22) $$s\left( t \right) = \left\{{\matrix{ {\hskip-7pc}{\displaystyle{1 \over 2}a{t^2}}  {0\ {\leq }\ t\ \ {\leq}\ \ {t_a}} \cr {\hskip-6pc}{vt - \displaystyle{{{v^2}} \over {2a}}}  {{t_a} \lt t\ \ {\leq}\ \ T - {t_a}} \cr {\displaystyle{{2avT - 2{v^2} - {a^2}{{\left( {t - T} \right)}^2}} \over {2a}}}  {T - {t_a} \lt t\ \ {\leq}\ \ T} \cr {}  {} \cr } } \right.$$

A legged robot is a floating base system that needs to plan its four toes and body simultaneously. First, as shown in Eq. (23), is the trajectory planning of feet. Between every two footholds, the robot use elliptical trajectory transitions. Second, the body’s trajectory is determined by the number of steps and the position of the foothold shown in Eq. (24). Here *L* is the step length of forwarding direction; *W* is the step length of right and left directions.

(23) $$\left\{{\matrix{{\hskip-0.6pc}{{x_{leg}} = {x_{pre}} + L + Lcos\left( {\pi - s\left( t \right)} \right)}  {0\ {\leq}\ t\ {\leq}\ T} \cr {{\hskip-3.8pc}{y_{leg}} = H\ sin\left( {\pi - s\left( t \right)} \right)}  {0\ {\leq}\ t\ {\leq}\ T} \cr {{z_{leg}} = {z_{pre}} + W + Wcos\left( {\pi - s\left( t \right)} \right)}  {0\ {\leq}\ t\ {\leq}\ T} \cr {}  {} \cr } } \right.$$

(24) $$\left\{{\matrix{ {{x_{body}}}  { = \left\{ {\matrix{ {{x_{prepos}} + \displaystyle{{L{t^2}} \over {4{T^2}}}} \cr {{x_{prepos}} + \displaystyle{L \over 4} + \displaystyle{L \over {2T\left( {t - T} \right)}}} \cr {{x_{prepos}} - \displaystyle{{L{{\left( {t - \displaystyle{{2n - 1} \over {{T^2}}}} \right)}^2}} \over {{T^2}}} + L\left( {n - \displaystyle{1 \over 2}} \right)} \cr {} \cr } } \right.} \cr {{\hskip-13.6pc}{y_{body} = 0}} \cr {{z_{body}}}  { = \left\{ {\matrix{ {{z_{prepos}} + \displaystyle{{W{t^2}} \over {4{T^2}}}} \cr {{z_{prepos}} + \displaystyle{W \over 4} + \displaystyle{W \over {2T\left( {t - T} \right)}}} \cr {{z_{prepos}} - \displaystyle{{L{{\left( {t - \displaystyle{{2n - 1} \over {{T^2}}}} \right)}^2}} \over {{T^2}}} + W\left( {n - \displaystyle{1 \over 2}} \right)} \cr {} \cr } } \right.} \cr {}  {} \cr } } \right.$$

**Figure 9:**
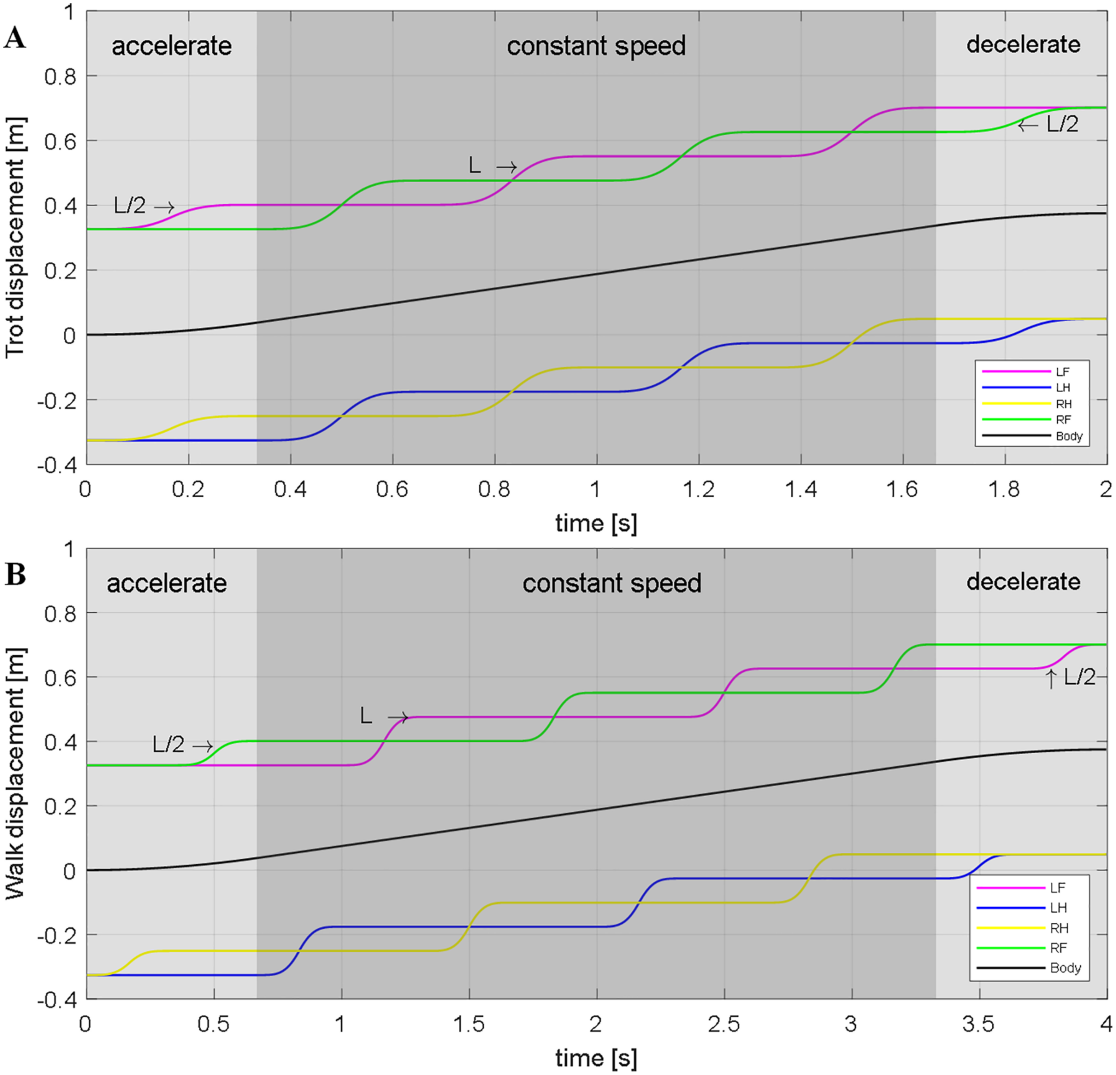
Trajectory of (A) Trot and (B) Walk gaits. *L* is the step length.

Figure 9 shows the trajectory of leg and body in Cartesian space when robot walking with trot and walk gait. These trajectories are expressed in-ground coordinate systems. We can use Eq. (25) to transfer it from *G* frame to *Leg* frame. Then the joint rotation angle was calculated by the inverse kinematics. Sent the angle to the corresponding motor can make the robot walking with a planned gait.

(25) $$^L\vec x = {^L}P_B^B{P_G}{\left( t \right)^G}\vec x$$

### Kinematic simulation

**Figure 10:**
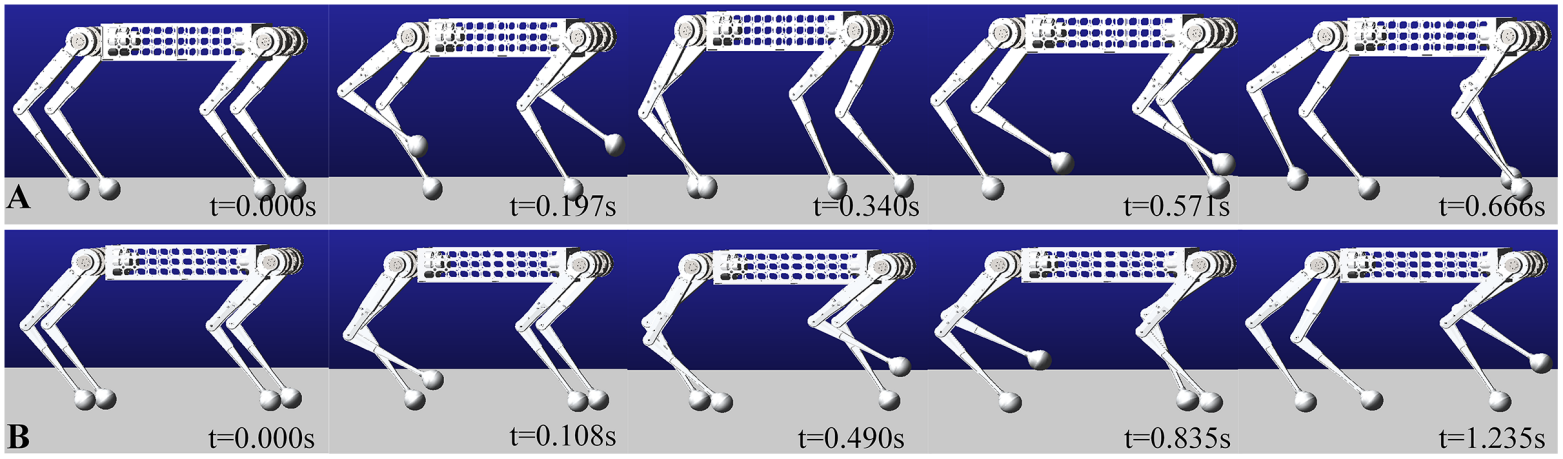
Trot and Walk simulation in ADAMS. Walking the same distance will save half the time attrot gait. (A) Simulation in trot gait. (B) Simulation in walk gait.

This paper use ADAMS to verify the kinematic and dynamic models. The physics engine of ADAMS has high computational precision. It can accurately reflect the ground reaction force of the robot and joint output torque. It can provide a theoretical basis for the mechanism design and hardware selection. The body and links are made of aluminum alloy in the simulation, close to the prototype used. The only difference is that the prototype has actuators, IMU, force sensors, batteries but look at them as a whole with the body in the simulation. We simplified the model and equivalently added all of these masses to body mass, ignoring the influence of its inertia. Figure 10 shows the schematic diagram of a quadruped robot walking in the trot and walk gait.

### Dynamic simulation

**Figure 11:**
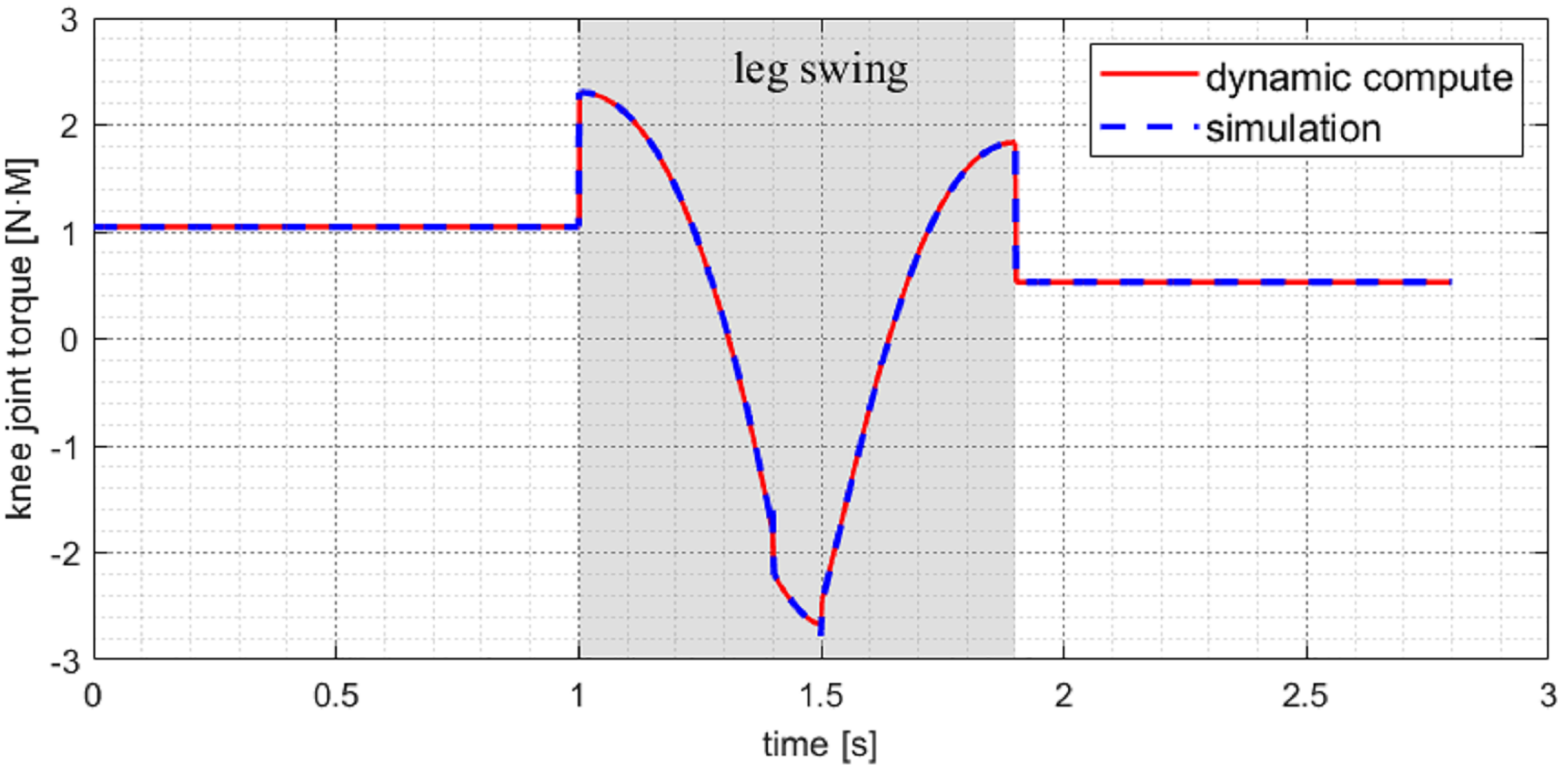
Knee joint torque of swing leg in walk gait under simulation. The model calculated results are extremely close to the simulation.

When the robot is in motion with a walking gait, three legs are always in the support phase and another leg in the swing phase. At this time, the body is stationary relative to the ground. Figure 11 shows the torque curve of the knee joint of the swing leg during the switching between the swing phase and the support phase. It can be seen that the calculation results of the dynamic model are the same as the simulation results. It is shown that the dynamic model has high computational accuracy and veracity under this topology structure. In addition, the torque of the swing leg’s joint will suddenly change at the moment of switching. That is due to the disappearance and appearance of external forces, leading to a sudden change in joint acceleration.

### Experiment in prototype

Our experiments were conducted on “XiLing”, a high dynamic response quadrupedal robot used an embedded PC with an Intel Core i7 4500U, running Ubuntu 16.04 (Linux-4.9.90 kernel) with Xenomai3 patch. The level communicates at 1 kHz over EtherCAT and control signals are generated in a 1 ms control loop that runs on a dedicated onboard industrial PC. The robot is driven by 12 identical motors, which are actuated by Elmo driver and are powered by a 48 V lithium battery. Table 1 shows motor performance parameters, with a continuous torque of 40 N·M and maximum torque of 108 N·M. In the prototype, the shank is driven by a gear belt with a reduction ratio of 1:2. The torque bearing capacity of the knee joint can reach 216 N·M, which completely satisfied the maximum torque requirements of the simulation. In other periods, the joint force is below 40 NM, which is lower than the continuous output torque of the motor.

**Table 1:** Motor parameters.

Parameters	Value	Units
Mass	0.96	kg
Gear ratio	20	–
Continuous torque	40	N·M
Max torque	108	N·M
Max joint speed	350	RPM

**Figure 12:**
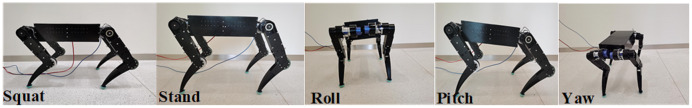
Various postures of the prototype in standing.

**Figure 13:**
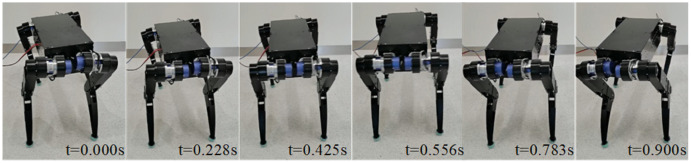
Yaw series with yaw angle take 0.9 s from 15° to −15°.

**Figure 14:**
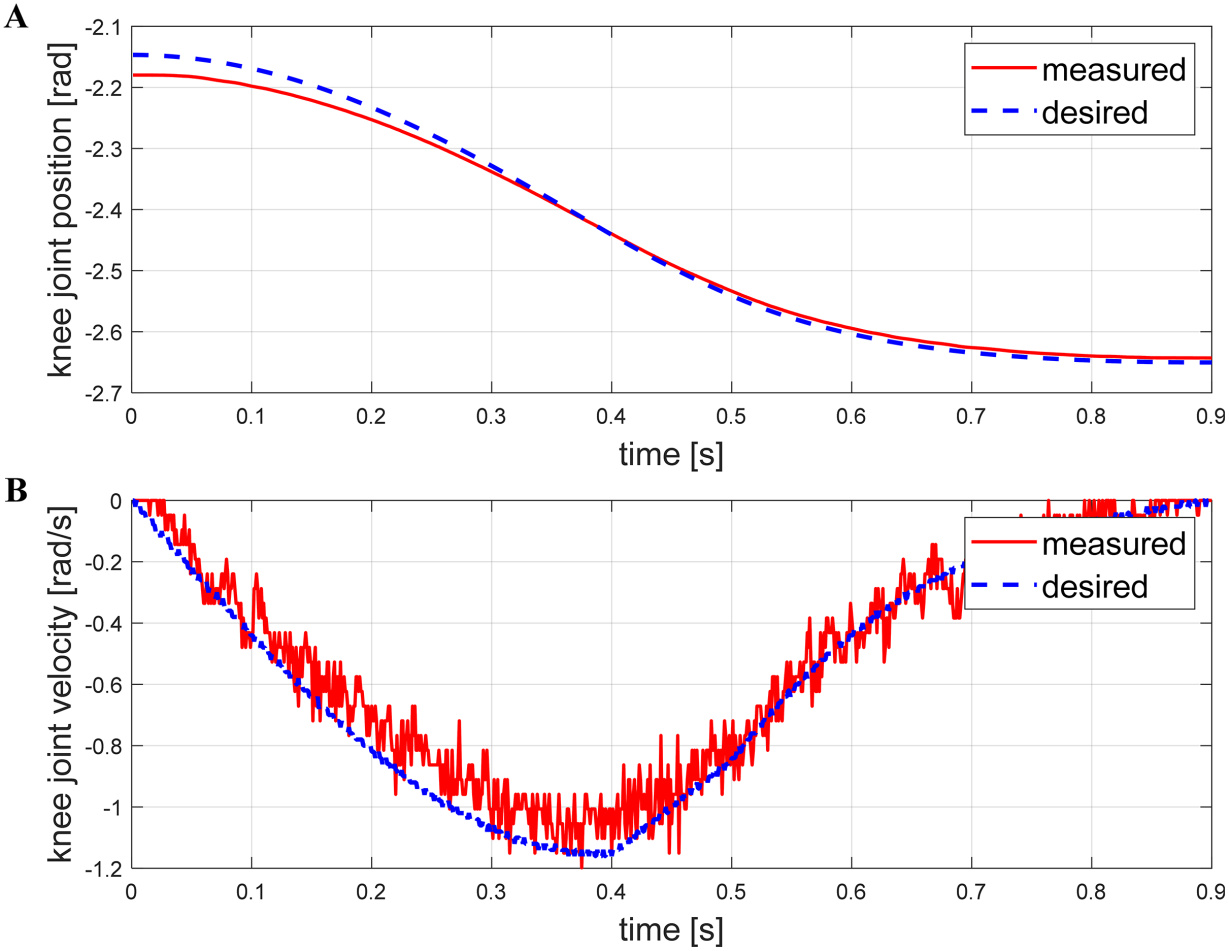
Track tracking effect of the knee joint while yawing. The desired position (A) and velocity (B) are presented with the measured position and velocity at the knee angle. The results show that the actual system follows the given commands well.

The robot can quickly switch any posture in its workspace while standing. Figure 12 shows the basic movements such as roll, pitch, yaw. Respectively rotation angles of them are *±*5°, *±*10°, ±20°. The trapezoidal curve is used to complete the planning during the movement, and the time of the switching process can be adjusted by modifying the acceleration and the maximum speed. Figure 13 illustrates a snapshot of the yaw angle changing from −15° to 15° within 0.9 s. Figure 14 shows the tracking curve of knee position and velocity during this movement. With 1,000 points interpolated per second, both allow for fast and accurate tracking. It is worth noting that the measured velocity curve has burrs because of the gear backlash and the friction between the belt and pulley. Burrs can be eliminated by choosing more precise gears and optimizing the planning control algorithm. We will prioritize this problem in the next step.

## Conclusion and Future Work

This paper presented whole-body kinematic and dynamic modeling methods for quadruped robots based on screw theory. Compared with traditional LIP or centroidal models, 10-dimensional mass and inertia of all parts are considered, which means higher precision. We divided the model into three phases: stand, walk, bound. Controller and plan strategies based on these models are proposed under each state. The motor torque curves of different gaits are calculated and compared to simulating software, which shows that computing and simulating results are identified. Prototype experiments of the standing phase are provided, and it turned out that the measured curves are very close to theoretical ones. In the future, we will focus on dynamic parameter identification. Contact models should also be improved *via* considering friction to get better results under slipping conditions.

## Supplemental Information

10.7717/peerj-cs.821/supp-1 Supplemental Information 1 Forward in trot gait.

10.7717/peerj-cs.821/supp-2 Supplemental Information 2 The raw data and code of the figure.

10.7717/peerj-cs.821/supp-3 Supplemental Information 3 A video of the prototype yaw.

10.7717/peerj-cs.821/supp-4 Supplemental Information 4 A video of the prototype pitch.

10.7717/peerj-cs.821/supp-5 Supplemental Information 5 A video of the prototype roll.

10.7717/peerj-cs.821/supp-6 Supplemental Information 6 Symbols in kinematics and dynamics.

10.7717/peerj-cs.821/supp-7 Supplemental Information 7 Inertia parameters of link in the center of mass coordinate system.

## Additional Information and Declarations

### Data Availability

The following information was supplied regarding data availability: The raw data and code for all curves are available in the Supplemental File.
