# Supplementary material for: Whole-body kinematic and dynamic modeling for quadruped robot under different gaits and mechanism topologies
Source: PeerJ Comput Sci. 2021 Dec 16;7:e821. doi: 10.7717/peerj-cs.821 (PMC8725662; doi:10.7717/peerj-cs.821)
Supplement: Supplemental Information 6 [file peerj-cs-07-821-s006.docx]

**Appendix I. Symbols in kinematics and dynamics**

| **Symbol** | **Description** |
| --- | --- |
|  | The distance between the origin of the front leg and the back leg |
|  | The distance between the origin of the left leg and the right leg |
|  | Body high with stand |
|  | Link length of hip |
|  | Upper leg length |
|  | Lower leg length |
|  | The inertia of the leg in B coordinate frame, where =1,2,3,4 is the leg and =1,2,3 is the joint in leg  |
|  | In the symbol below, the =1,2,3,4 is the leg and =1,2,3 is the joint in leg  |
|  | Screw axis of  joint of  leg |
|  | Initial velocity screw on B coordinate frame |
|  | Velocity screw on B coordinate frame at any time |
|  | Initial constraint force screw of joint on B coordinate frame |
|  | Constraint force screw of joint on B coordinate frame at any time |
|  | Initial constraint force screw of motor on B coordinate frame |
|  | Constraint force screw of motor on B coordinate frame at any time |
|  | Input angle of joint  of leg  |
|  | Input velocity of joint  of leg  |
|  | Accelerate of joint  of leg  |
|  | The constraint matrix of all the members |
|  | Homogeneous transformation matrix |
|  | Velocity screw conversion matrix |
|  | Force screw conversion matrix |
