# Supplementary material for: Whole-body kinematic and dynamic modeling for quadruped robot under different gaits and mechanism topologies
Source: PeerJ Comput Sci. 2021 Dec 16;7:e821. doi: 10.7717/peerj-cs.821 (PMC8725662; doi:10.7717/peerj-cs.821)
Supplement: Supplemental Information 7 [file peerj-cs-07-821-s007.docx]

**Appendix B-Inertia parameters of link in the center of mass coordinate system**

| Link | m | *I_xx_* | *I_yy_* | *I_zz_* | *I_xy_* | *I_xz_* | *I_yz_* |
| --- | --- | --- | --- | --- | --- | --- | --- |
| Body | 15.100717743 | 0.68976308 | 0.612989762 | 0.1389151407 | 0 | 0 | 0 |
| LF-*l*_1_ | 2.3523814491 | 1.04808098E-02 | 8.88259037E-03 | 4.26675463E-03 | 0 | 0 | 0 |
| LF-*l*_2_ | 0.5868045476 | 6.97388713E-03 | 6.89325855E-03 | 5.60653084E-04 | 0 | 0 | 0 |
| LF-*l_3_* | 1.0111367876 | 1.19559334E-02 | 1.16566507E-02 | 5.88197758E-04 | 0 | 0 | 0 |
| LH-*l*_1_ | 2.3523814491 | 1.04808098E-02 | 8.88259037E-03 | 4.26675463E-03 | 0 | 0 | 0 |
| LH-*l*_2_ | 0.5868045476 | 6.97388713E-03 | 6.89325855E-03 | 5.60653084E-04 | 0 | 0 | 0 |
| LH-*l*_3_ | 1.0111367876 | 1.19559334E-02 | 1.16566507E-02 | 5.88197758E-04 | 0 | 0 | 0 |
| RH-*l*_1_ | 2.3523814491 | 1.04808098E-02 | 8.88259037E-03 | 4.26675463E-03 | 0 | 0 | 0 |
| RH-*l*_2_ | 0.5868045476 | 6.97388713E-03 | 6.89325855E-03 | 5.60653084E-04 | 0 | 0 | 0 |
| RH-*l*_3_ | 1.0111367876 | 1.19559334E-02 | 1.16566507E-02 | 5.88197758E-04 | 0 | 0 | 0 |
| RF-*l*_1_ | 2.3523814491 | 1.04808098E-02 | 8.88259037E-03 | 4.26675463E-03 | 0 | 0 | 0 |
| RF-*l*_2_ | 0.5868045476 | 6.97388713E-03 | 6.89325855E-03 | 5.60653084E-04 | 0 | 0 | 0 |
| RF-*l*_3_ | 1.0111367876 | 1.19559334E-02 | 1.16566507E-02 | 5.88197758E-04 | 0 | 0 | 0 |
